# Comprehensive mastocytosis data analysis from a single center

**DOI:** 10.1186/s12885-022-10498-3

**Published:** 2023-01-24

**Authors:** Tarık Onur Tiryaki, Sıdıka Gülkan Özkan, Simge Erdem, Aynur Dağlar Aday, İpek Yönal Hindilerden, Aslı Gelincik, Can Baykal, Gülçin Yegen, İbrahim Öner Doğan, Nesimi Büyükbabani, Meliha Nalçacı, Akif Selim Yavuz

**Affiliations:** 1 Faculty of Medicine, Department of Internal Medicine, Division of Hematology, Istanbul University, Istanbul, Turkey; 2 Faculty of Medicine, Department of Internal Medicine, Division of Medical Genetics, Istanbul University, Istanbul, Turkey; 3 Faculty of Medicine, Department of Internal Medicine, Division of Allergy, Istanbul University, Istanbul, Turkey; 4 Faculty of Medicine, Department of Dermatology, Istanbul University, Istanbul, Turkey; 5 Faculty of Medicine, Department of Pathology, Istanbul University, Istanbul, Turkey

**Keywords:** Mastocytosis, An orphan disease, Comprehensive analysis, Cytoreductive therapy, Extended clinical Spectrum

## Abstract

Mastocytosis is a very rare disorder and is divided into three prognostically distinct variants by World Health Organization: Cutaneous mastocytosis (CM), systemic mastocytosis (SM), and mast cell sarcoma or localized mast cell (MC) tumors. The wide range of complaints may cause patients to consult various clinics, with resulting mis- or underdiagnosis. Therefore, cooperation between different subspecialties is of paramount importance. In this article, we have compiled 104 adult mastocytosis cases diagnosed and followed in our Hematology and other clinics. 86 (82.7%) of 104 patients had systemic mastocytosis. Osteoporosis, disease-related complications, and secondary malignancies are important topics in this group. We know that indolent form has great survival. But smoldering or aggressive mastocytosis has a poor prognosis. CM and indolent SM have a significantly better prognosis compared to aggressive SM (*p* < 0.001). We found that the presence of more than 25% of mast cells in the bone marrow, the presence of concomitant marrow dysplasia, and the presence of disease-related complications affect survival (*p* < 0.001). In addition to the WHO classification, the IPSM scoring system is indicative of the prognosis in this rare disease.

## Introduction

Mastocytosis is a rare disease with diverse presentation and variable prognosis, characterized by clonal mast cell proliferation. It is considered to be an orphan disease, and there is the limited number of epidemiological studies. In a Danish study, the overall incidence of systemic mastocytosis (SM) was 0.9 per 100,000 per year, and more than 80% of these patients were diagnosed with indolent systemic mastocytosis (ISM) [1]. In another study, the prevalence of ISM in the adult population in the Netherlands has been estimated to be 13 cases per 100,000 inhabitants [2]. In a study from Germany, the incidence and prevalence of patients with advanced systemic mastocytosis were 0.9 and 7 per 1 million inhabitants, respectively [3]. In a Swedish study, the incidence was 0.77/100.000 people per year [4]. It is seen at similar frequencies in different populations with an increase in awareness.

In patients with mastocytosis, various symptoms and signs may be observed due to mediators released from mast cells and organ dysfunction due to mast cell infiltration. These mediators can cause dermatological (pruritus, flushing, Darier sign), gastrointestinal (abdominal cramping, nausea, vomiting, diarrhea), respiratory and cardiovascular system (anaphylaxis, hypotension, syncope, dyspnea), musculoskeletal (osteopenia, osteoporosis, pathological bone fractures), neurological and psychiatric complaints (depression, neurocognitive impairment). The diagnosis can be missed due to this diversity. Regardless of the subtype, patients may complain of mediator symptoms and life-threatening allergies.

According to the World Health Organization (WHO) 2016 update, mastocytosis is divided into three major groups: Cutaneous mastocytosis (CM), systemic mastocytosis (SM), and mast cell sarcoma or localized mast cell (MC) tumors. Diagnostic criteria are defined in Table 1.

**Table 1:** Systemic mastocytosis criteria

Major Criterion^a^	
-Multifocal dense mast cells in the bone marrow or other extra-skin organs infiltration (presence of more than 15 mast cells in aggregates)	
Minor Criteria^a^	
-Abnormal mast cells in bone marrow or other extra-skin organs (>%25)	
-Presence of Asp-816-Val c-KIT mutation in extra skin organs^b^	
-CD2 and CD25 positivity in bone marrow mast cells	
-Serum tryptase level > 20 ng / mL (not applied in the presence of hematological clonal disease)	

^a^The combination of 1 major and 1 minor criteria, or at least three minor criteria coexistence is sufficient for diagnosis

^b^Other activating mutations at codon 816 are also valid

In the updated WHO classification, SM is divided into different subgroups: Indolent SM (ISM), smoldering SM (SSM), SM-AHN (SM with an associated hematologic [non-MC lineage] neoplasm), aggressive SM (ASM), mast cell leukemia (MCL) according to the burden of disease, percent of mast cell infiltration, accompanying signs of organ damage (Table 2). MCL is diagnosed with the presence of ≥20% mast cells in the bone marrow smear.

**Table 2:** B and C findings in SM

**B ‘Burden of disease’ findings**^**a**^	
- Bone marrow biopsy showing > 30% infiltration by MCs and serum tryptase level > 20 ng/ml - Myeloproliferation or signs of dysplasia in non–MC lineage(s), no prominent cytopenias; criteria for AHN not met - Hepatomegaly and/or splenomegaly without impairment of organ function and/or lymphadenopathy on palpation/imaging (> 2 cm)	
**C ‘Cytoreductive therapy requiring’ findings**^**b**^	
- Cytopenia(s): ANC < 1000/μL, Hb < 10 g/dL, or platelets < 100,000/μL - Hepatomegaly with impairment of liver function, ascites, and/or portal hypertension - Palpable splenomegaly with hypersplenism - Hepatomegaly with hypoalbuminemia and weight loss from gastrointestinal tract MC infiltrates - Skeletal lesions: osteolyses and/or pathologic fractures - Life-threatening organ dysfunction caused by mast cell infiltration	

^a^Smoldering SM is defined ≥2 ‘B’ findings, absence of ‘C’ findings or an AHN

^b^*ASM*; ≥*1 C finding/s*

The prognosis, symptoms, and treatment vary among patients depending on the disease variant, the presence of an additional hematologic neoplasm as well as the presence of comorbidities [5].

In this study, we aimed to assess the current data and clinical findings of 104 adult patients diagnosed with cutaneous and/or systemic mastocytosis in our clinic, Istanbul University, Faculty of Medicine which is a center of excellence of the European Competence Network on Mastocytosis.

## Patients and methods

### Patients

This study was approved by the institutional ethics committee (Istanbul University, Istanbul Faculty of Medicine Ethics Committee, Istanbul/Turkey). The procedures followed were in accordance with the Declaration of Helsinki 1975, as revised in 2000, and patients had provided written informed consent. The patients and the data were collected retrospectively at the Istanbul Faculty of Medicine, Division of Hematology. The primary objective of the study was to evaluate the demographic features, clinical and laboratory findings at the diagnosis, treatments and complications during the follow up in our center. The measurement of tryptase was made by the Fluoro-Enzymatic Immunoassay (FEIA) method. KIT D816V mutation was analyzed with a highly sensitive real time polymerase chain reaction (RT-PCR) assay [1]. Bone findings were investigated with direct radiography and dual energy X-ray absorptiometry (DEXA). T scores measured by DEXA (Osteoporosis: T score, below the mean of young healthy adults less than − 2.5, Osteopenia: T-score ranging from − 1 to − 2.5) and the presence of sclerotic or lytic lesions were evaluated with direct radiography. Survival was evaluated with WHO classification and International Prognostic Scoring System in Mastocytosis (IPSM) [2]. A total of 104 patients with mastocytosis were enrolled; they had been diagnosed at the Division of Hematology according to the WHO criteria [3]. Patients were excluded if they did not fulfill WHO criteria for mastocytosis and if they did not attend follow-up regularly. Treatment responses in ASM were evaluated based on the International Working Group-Myeloproliferative Neoplasms Research and Treatment (IWG-MRT) & European Competence Network on Mastocytosis (ECNM) consensus response [4]. The disappearance of mast cell infiltration in the affected organs, decrease in serum tryptase level below 20 ng/ml, disappearance of C findings and peripheral blood count remission were defined as complete response (CR). Without complete response, reduction by ≥50% in neoplastic MCs in the affected tissue, reduction of serum tryptase level by ≥50% and resolution of one or more biopsy-proven or suspected SM-related organ damage were considered as a partial response (PR). Clinical improvement (CI) was defined as the presence of at least one of the hematological or non-hematological response criteria that did not meet the CR, PR or progressive disease (PD). Deterioration in the prior laboratory abnormality, decrease in albumin (increase in severity or decrease more than 0.5 g/dl), new transfusion dependence or increase in the average transfusion frequency, increase in spleen and liver size were considered as PD. Patients who did not meet these response criteria were considered as stable disease (SD).

In IPSM scoring, the nonadvanced group was classified as intermediate risk group 1 and 2 according to age (> 60) and serum alkaline phosphatase (> 100 u/l) value. Patients without these risk factors were defined as the low risk group. In patients with advanced systemic mastocytosis, age ≥ 60 years, tryptase ≥125 ng/mL, leukocytes ≥16 × 10^3^/μL, hemoglobin ≤11 g/dL, platelets ≤100 × 10^3^/μL and skin involvement are independent prognostic factors for overall survival. Patients who have no risk factors are grouped in advanced systemic mastocytosis 1 (AdvSM-1), those with one risk factor in AdvSM-2, individuals with two or three risk factors in AdvSM-3, and patients with four or five risk factors in AdvSM-4 subgroup [2].

### Statistical analysis

All analyses were performed on SPSS v21 (SPSS Inc., Chicago, IL, USA). Shapiro-Wilk test was used to determine whether variables are normally distributed. Normally distributed variables were analyzed with the independent samples t test or one-way analysis of variances (ANOVA) depending on count numbers of groups. Pairwise comparisons were performed with the Tukey test or Tamhane test depending on homogeneity of variances. Non-normally distributed variables were analyzed with the Mann Whitney U test or Kruskal Wallis test depending on the count numbers of groups. Pairwise comparisons were performed with the Bonferroni correction method. Categorical variables were analyzed with the chi-square test or Fisher’s exact test. Survival times were calculated with the Kaplan-Meier method. Cox regression analysis (forward conditional method) was performed to determine significant prognostic factors of the mortality. Two-tailed *p*-values of less than 0.05 were considered statistically significant.

## Results

We included 104 patients (49 females and 55 males) into our study. Median age at the start of symptoms was 35 (range 14–86) and median age at diagnosis was 41 (range 18–87). Mean follow-up period was 50.87 ± 49.43 (range 1–256) months and 16 (15.38%) patients died due to different reasons (Table 3).

**Table 3:** Summary of patients characteristics

Gender (***N*** = 104)	n, %
Female	49 (47.12%)
**Median age at onset**	35 (14–86)
**Median age at diagnosis**	41 (18–87)
**Follow-up status**	
Alive	67 (64.42%)
Lost to follow-up	21 (20.19%)
Dead	16 (15.38%)
**Reason of death (*n*** **= 16)**	
Malignancy	2 (12.50%)
Malignancy & Infection	1 (6.25%)
Infection	3 (18.75%)
Transplant releated mortality	2 (12.50%)
AML transformation	2 (12.50%)
Cardiovascular disease	5 (31.25%)
Unknown	1 (6.25%)
**Follow-up time, months**	50.87 ± 49.43 (1–256)

Eighteen (17.3%) patients had CM and 86 (82.7%) patients were diagnosed with systemic mastocytosis. In this systemic mastocytosis group, 55 (52.9%) patients had ISM, 14 (13.5%) patients had SM-AHN, 6 (5.8%) patients had SSM, 6 (5.8%) patients had ASM, and 5 (4.8%) patients had MCL.

In our series, the most common first symptom was skin lesions (63.5%).The other common first symptom was anaphylaxis (19.2%). The most common symptoms were skin symptoms (76%) (skin lesions associated with mast cell infiltration and other skin symptoms (pruritus, flushing), hepatomegaly (32.7%), anaphylaxis (28.9%) and bone symptoms (28.9%). Seventy three (79.4%) patients had normal x-ray and 18 (19.4%) patients had normal DEXA. Different complications occurred in 18 (17.5%) patients (Table 4). BM mast cell percent was above 25% in 14 (16.3%) patients. Eighty-six (82.7%) patients had mast cell aggregates, the most common types were patchy-scattering (37.2%) and nodules (34.9%). Eighthy five (81.7%) patients were BM tryptase positive, 85 (82.5%) patients were BM CD117 positive, 24 (55.8%) patients were BM CD30 positive, 78 (85.7%) patients were C-KIT D816V positive and 15 (14.4%) patients had dysplasia.

**Table 4:** Summary of disease characteristics

**Diagnosis (*N*** **= 104)**	**n, %**
Cutaneous mastocytosis	18 (17.31%)
Systemic mastocytosis	86 (82.69%)
*Indolent SM*	55 (52.88%)
*SM-AHN*	14 (13.46%)
*Smoldering SM*	6 (5.77%)
*Aggressive SM*	6 (5.77%)
*Mast cell leukemia*	5 (4.81%)
**SM-AHN type (*n*** **= 14)**	**n, %**
ASM-CMML	2 (14.29%)
ASM-MF	2 (14.29%)
ASM-MM	1 (7.14%)
ISM-CML	1 (7.14%)
ISM-CMML	1 (7.14%)
ISM-ET	1 (7.14%)
ISM-MGUS	1 (7.14%)
ISM-MZL	1 (7.14%)
MCL-CMML	1 (7.14%)
MCL-MDS	1 (7.14%)
SSM-MGUS	1 (7.14%)
SSM-PV	1 (7.14%)
**Skin involvement, %**	33.44 ± 28.00 (0–91)
**First symptom**	**n (%)**
Skin lesion	66 (63.46%)
Anaphylaxis	20 (19.23%)
Constitutional symptom	7 (6.73%)
Abdominal pain	2 (1.92%)
Dyspnea	2 (1.92%)
Flushing	2 (1.92%)
Bone pain	2 (1.92%)
Weakness	1 (0.96%)
Itching	1 (0.96%)
Asymptomatic	1 (0.96%)
**BM mast % (*N*** **= 86)**	**n (%)**
< 25%	72 (83.72%)
> 25%	14 (16.28%)
**BM mast cell aggregate**	86 (82.69%)
Scattering	14 (16.28%)
Patchy-scattering	32 (37.21%)
Nodule	30 (34.88%)
Diffuse	10 (11.63%)
**BM dysplasia**	**n (%)**
No	89 (85.58%)
Rarely, dysplasia <%10	15 (14.42%)
**Skin symptoms**	79 (75.96%)
**Hepatomegaly**	34 (32.7%)
Associated with hepatosteatosis	24 (23.08%)
Associated with mast cell disease	10 (9.62%)
**Anaphylaxis**	30 (28.85%)
**Bone symptoms**	30 (28.85%)
**GI tract symptoms**	22 (21.15%)
**Splenomegaly**	18 (17.31%)
**Constitutional symptom**	15 (14.42%)
**CVS symptoms**	13 (12.50%)
**Lymphadenomegaly**	13 (12.50%)
**Ascites**	8 (7.69%)
**Portal hypertension**	7 (6.73%)
**X-ray (*N*** **= 92)**	**n (%)**
Normal	73 (79.35%)
Osteopenia	4 (4.35%)
Osteosclerosis	8 (8.70%)
Lytic lesion	4 (4.35%)
Sclerotic and lytic lesions	3 (3.26%)
**DEXA (*N*** **= 93)**	**n (%)**
Normal	18 (19.35%)
Osteopenia (> 2.5 and < −1.0)	50 (53.76%)
Osteoporosis (< −2.5)	18 (19.35%)
Osteosclerosis	7 (7.53%)
**Complications**^**a**^	18 (17.48%)
Portal hypertension	7 (6.80%)
Duodenal perforation	1 (0.97%)
GI bleeding	4 (3.88%)
Compression fracture	4 (3.88%)
Pleural effusion	2 (1.94%)
Portal vein thrombosis	1 (0.97%)
Malignancy	6 (5.83%)

^a^Some patients had multiple complications

*CMML* chronic myelomonocytic leukemia, *MF* myelofibrosis, *MM* multiple myeloma, *CML* chronic myeloid leukemia, *ET* essential thrombocytosis, *MGUS* monoclonal gammopathy of undetermined significance, *MDS* myelodysplastic syndrome, *MZL* primary cutaneous marginal zone B-cell lymphoma, *PV* polycythemia vera

Hepatomegaly associated with mast cell disease (*p* = 0.026) and splenomegaly (*p* = 0.006) percentages were significantly higher in the patients with positive BM CD30 than in patients with negative BM CD30. There were no significant differences between patients with positive and negative BM CD30 regarding gender, age at the start of the symptoms, age at diagnosis, x-ray findings, DEXA results, skin involvement, lymphadenomegaly (LAM), cardiovascular symptoms, GI tract symptoms, skin symptoms, ascites, portal hypertension, anaphylaxis, B symptoms, bone symptoms or mortality.

The most common treatments were H1 antagonists (94.23%) and H2 blockers (92.30%). Gastrointestinal complaints of 61 patients (69.32%) regressed with H2 blocker treatment.

We observed partial response in 2 patients (22.2%) and CI in 3 patients (33.3%) with cladribine treatment.

No response was obtained with IFN in 6 patients (5.77%).

Imatinib treatment was given to 5 (4.81%) patients. Four of these patients did not have the D816V KIT mutation and adequate response could not be obtained in all of them. One patient received imatinib due to the diagnosis of concomitant CML.

We applied dasatinib treatment to a limited number of patients (*n* = 2) without success.

We administered azacitidine to a patient who was diagnosed with SM-AHN (MCL-MDS) and observed no response.

Seventeen (16.4%) patients received midostaurin. CI was achieved in 9 patients and PR was achieved in 8 patients. Cytopenia and constitutional symptoms improved with midostaurin treatment. This treatment was discontinued in 3 patients due to intolerance or adverse reactions (two patients had refractory nausea and 1 patient toxic hepatitis). Since tissue biopsy was not repeated in all patients who received treatment, the presence of complete response could not be evaluated. Currently, 5 patients with partial response are still on follow-up.

We applied avapritinib treatment in one patient who had toxic hepatitis with midostaurin. In the third month of the treatment, response was observed in skin lesions due to mast cell infiltration and constitutional symptoms decreased. The patient is still receiving avapritinib therapy.

Allogeneic stem cell transplantation was applied to 4 (3.9%) patients. Three of these patients were diagnosed with SM-AHN (ASM-CMML in 2 patients, ASM–myelofibrosis (ASM-MF) in 1 patient). One patient with the diagnosis of ASM was responsive to midostaurin treatment and an allogeneic stem cell transplantation was performed due to transformation to AML, but died due to relapsing leukemia. The patient with ASM-CMML died due to infection in the early period. The other patient with ASM-CMML died due to leukemia, too. One patient with ASM-MF is still on follow-up (Table 5).

**Table 5:** Summary of treatment characteristics (During follow up)

**Mediator release treatment**	**n (%)**
***Antihistamine (H1 blocker)***	98 (94.23%)
***H2 blocker***	96 (92.30%)
***Montelukast***	12 (11.54%)
***Omalizumab***	11 (10.58%)
**Cytoreductive treatment**	**n (%)**
***IFN***	6 (5.77%)
Response	
SD	4 (66.7%)
PD	2 (33.3%)
***Cladribine***	9 (8.65%)
Response	
SD	4 (44.5%)
PR	2 (22.2%)
CI	3 (33.3%)
***Imatinib***^b^	5 (4.81%)
Response	
SD	3 (60.0%)
PD	1 (20.0%)
***Dasatinib***	2 (1.92%)
Response	
SD	2 (100.0%)
***Midostaurin***	17 (16.35%)
Response	
CI	9 (52.94%)
PR	8 (47.06%)
***Azacitidine***^**a**^	1 (0.96%)
***Avapritinib***	1 (0.96%)
Response	
CI	1 (100.00%)
***Allogeneic stem cell transplantation***	4 (3.85%)
**Cytoreductive Treatment Line**	
0	79 (75.96%)
1	9 (8.65%)
2	7 (6.73%)
3	6 (5.77%)
4	1 (0.96%)

^a^One patient was treated with azacitidine for accompanying myelodysplastic syndrome

^b^One patient received imatinib therapy for CML

**Fig. 1:**
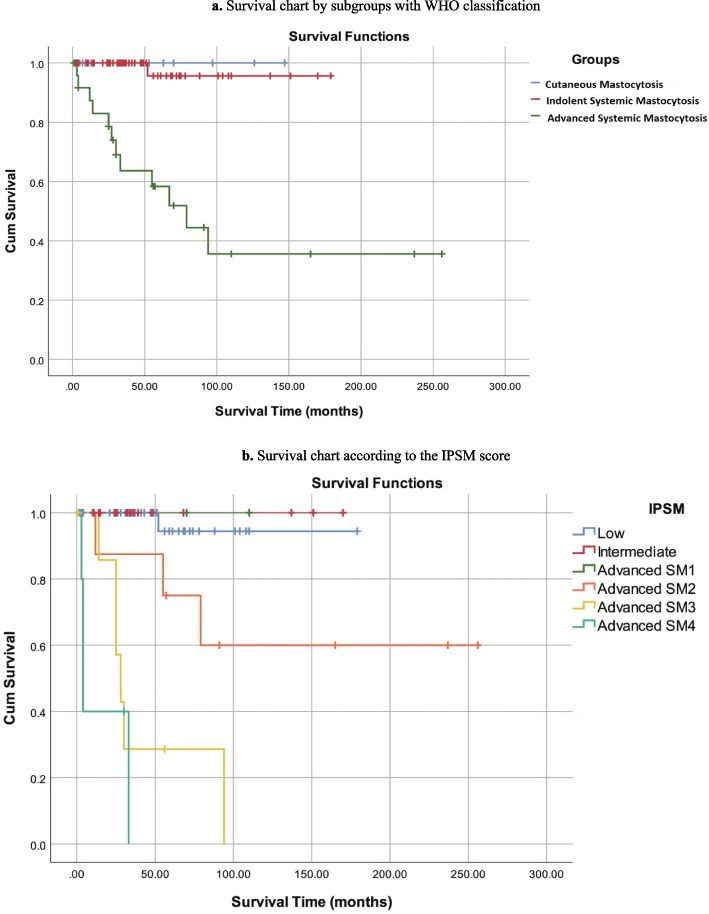
**A** Survival chart by subgroups with WHO classification. **B** Survival chart according to the IPSM score

A negative correlation was found between survival and mast cell infiltration rate in the bone marrow, accompanying dysplasia, and complications during the follow-up (*p* < 0.001) (Table 6). As expected, survival was better in patients with cutaneous mastocytosis (Table 6). Survival in advanced SM was the worst (Fig. 1a).

**Table 6:** Survival times (months) with Kaplan Meier method

	n	Exitus	Mean ± SE (95% CI)	***p***
**Overall survival**	104	16	194.32 ± 14.53 (165.84–222.80)	N/A
**Gender**				
Female	49	5	215.98 ± 17.77 (181.16–250.8)	0.093
Male	55	11	124.80 ± 13.82 (97.71–151.89)	
**X-ray**				
Normal	73	9	148.14 ± 10.29 (127.98–168.30)	0.368
Abnormal	19	4	177.60 ± 31.77 (115.32–239.88)	
**DEXA**				
Normal & Sclerosis	25	6	169.28 ± 30.01 (110.46–228.11)	0.418
< −1.00	68	10	182.09 ± 16.61 (149.54–214.63)	
**BM mast%**				
< 25%	73	8	194.58 ± 14.72 (165.73–223.43)	**< 0.001**
> 25%	15	8	58.17 ± 10.68 (37.25–79.10)	
**BM dysplasia**				
No	89	7	212.63 ± 15.62 (182.02–243.24)	**< 0.001**
Rarely dysplasia less than %10	15	9	50.59 ± 12.24 (26.59–74.58)	
**Complications**				
Absent	85	6	225.12 ± 12.18 (201.26–248.99)	**< 0.001**
Present	18	10	78.82 ± 17.67 (44.18–113.45)	
**WHO classification**				
CM	18	0	(1)	**< 0.001**
ISM	61	1	173.48 ± 5.40 (162.89–184.06)	
ASM	25	15	103.83 ± 23.13 (58.50–149.16)	
**IPSM score**				
Low risk non-advanced	44	1	171.94 ± 6.86 (158.51–185.38)^a^	**< 0.001**
Intermediate risk non-advanced	16	0	(1)	
Advanced SM-1	2	0	(1)	
Advanced SM-2	8	3	173.83 ± 37.40 (100.52–247.13)^ab^	
Advanced SM-3	8	6	44.29 ± 13.16 (18.49–70.08)^bc^	
Advanced SM-4	5	4	15.40 ± 7.42 (0.85–29.95)^c^	

(1) No statistics are computed because all cases are censored. Same letters denote the lack of statistically significant difference between groups

*SE* Standard error, *CI* Confidence interval

In the survival analyzes performed according to the IPSM scoring sytem, survival time was significantly longer in patients with low IPSM than in patients with AdvSM-3 and AdvSM-4. Survival time was significantly longer in the patients with AdvSM-2 than in the patients with advanced SM4 (*p* < 0.001) (Fig. 1b). Although there was a difference between AdvSM-2 and AdvSM-3, it could not be proven statistically. This was attributed to the low number of patients (Table 4).

## Discussion

Mast cells play a role in both adaptive and innate immune responses. In healthy individuals, small amounts of normal mast cells are present in the perisinusoidal or peritrabecular area within the BM [5]. Mastocytosis is a heterogeneous neoplasm with clonal expansion of mast cells in different organ systems, especially in the skin and hematopoietic tissue [6]. While normally mast cells do not express CD2, CD25 and CD35, these antigens become expressed in clonal mast cell disease [7].

With increase of awareness about the disease, real life demographic features can be obtained. Centers of excellence with hematologists, allergists, dermatologists, hematopathologists, dermatopathologists, gastroenterologists and geneticists play an important role in the diagnosis and follow-up of systemic mastocytosis. The most important step in the evaluation of SM in adults is bone marrow examination. In case of clinical suspicion, further investigation is required.

The distribution of mastocytosis patients in the data registry of ECNM until 2018 was as follows: 1570/2985 (52.5%) ISM, 523/2985 (17.5%) CM, 63/2985 (2.1%) SSM, 91/2985 (3%) ASM, 34/2985 (1.1%) MCL and 229/2985 (7.6%) SM-AHN [8]. The number of advanced systemic mastocytosis patients was higher in our series (6/104 (5.77%) ASM; 5/104 (4.81%) MCL and 14/104 (13.46%) SM-AHN) in comparison to ECNM data. This may be related to the fact that our center is a reference center for patients having C-findings.

Clonal mast cells typically carry somatic activating mutations that cause mast cells and their progenitors to gain function [9]. The most common of these mutations results from the substitution of aspartic acid with valine at position 816 (> 90% of cases) in the KIT proto-oncogene (KIT D816V) [3]. KIT D816V gene mutation was investigated with a highly sensitive allele-specific oligonucleotide - PCR (ASO-PCR) method in 91 of our patients, and it was found positive in 78 of them (85.7%).

In contrary to SM, CM is usually diagnosed in childhood and whereas systemic symptoms may be seen due to release of mediators the specific infiltration of mast cells is only limited to skin in this type of mastocytosis [3]. Morphologically, there are three types: maculopapular CM (urticaria pigmentosa) (UP), diffuse CM and mastocytoma. In many cases, the skin lesions disappear during puberty [10]. Skin lesions appearing in adults are usually a manifestation of SM but in some cases systemic involvement is not associated. The mean value of tryptase at diagnosis of 18 adult CM patients we followed in our clinic was found to be 5 ng/mL (4–7.5 ng/mL). Some common forms of CM may require systemic therapy, topical steroid, antihistamines and psoralen-UVA (PUVA) therapy. For skin lesions, omalizumab therapy may be beneficial, but usually the benefit is temporary [11, 12]. CM has a good prognosis with fewer complications [10]. CM has a better prognosis and no complications developed in the follow-up of this patient group in our clinic.

ISM is the most common form of systemic mastocytosis. In contrast to CM, ISM usually develops in adults. Bone marrow is always involved, but in typical ISM, the degree of bone marrow infiltration is very low. Bone marrow mastocytosis (BMM), a newly defined ISM subtype, also describes isolated marrow involvement without skin involvement [13]. In these patients, symptoms such as itching, flushing, diarrhea, abdominal cramps, vomiting, and meteorism may be observed. Antihistamines (H1 and H2 antihistamines) are used in treatment rather than cytoreductive drugs in this group. Proton pump inhibitors, cromolyn sodium and antacids can be used for gastrointestinal complaints. Vitamin D, calcium and bisphosphonate therapy can be used in patients with osteoporosis in these groups. We used H1 and H2 antihistamines and intermittent vitamin D level monitoring (every 3–6 months), bone density measurement (every 1–2 years) in this group. If necessary, vitamin D support and bisphosphonate therapy was administered.

While some of the SSM patients have a silent course, some may transform to aggressive mastocytosis or SM-AHN or MCL. The risk of progression depends on the type of SM. The cumulative probability of disease progression in ISM ranged from 1.7 ± 1.2% at 5–10 years to 8.4 ± 5% at 20–25 years [14]. In another study, only 1% of ISM patients evolved to SSM, ASM, or AML [15]. But 15% of patients with SSM showed progression (to ASM or AML) [15]. Leukemic transformation to acute myeloid leukemia [AML] occurs in 5–32% of ASM [16]. Transformation to AML was observed in 2 (8%) of the 25 ASM patients we followed up.

Diagnosis of SM-AHN can coexist with myeloid malignancies such as myeloproliferative neoplasms (MPNs), MDS/MPNs such as chronic myelomonocytic leukemia (CMML), atypical chronic myeloid leukemia, BCR-ABL1 negative, MDS/MPN unclassifiable, or AML, and less frequently accompanied by lymphoid malignancies (chronic lymphocytic leukemia, plasma cell neoplasms, or primary amyloidosis) [17]. Diagnosis may be difficult in SM-AHN, due to the underlying hematologic disease that may mask mast cells [18]. There are two different hypotheses in the etiology of SM-AHN. The first one is that the disease is caused by two different clones. The other is that it originates from a precursor with combined SM and AHN [19]. The most common hematological neoplasm associated with systemic mastocytosis is CMML which was the case in our group, too (4 of 14 patients) [3] (Table 4).

While the main symptoms of the disease occur due to mast cell degranulation in ISM patients, complaints may be observed due to mast cell infiltration and organ dysfunction in ASM. Physical and psychological stress factors, certain drugs and foods, insect bites, radiology contrast agents can cause mast cell degranulation. Allergic reactions, skin rashes are the most common symptoms of the indolent form [20].

In advanced systemic mastocytosis (advSM), cytopenia and organ dysfunctions may be the first form of presentation. While less skin findings are seen in advSM, hepatomegaly due to mast cell infiltration, lymphadenomegaly, GI system and cardiovascular system findings, ascites and portal hypertension are more common in this group. Various complications can be seen due to involvement of different organs. Portal hypertension, fragility fractures, duodenal perforation and gastrointestinal bleeding, pleural effusion, portal vein thrombosis, and solid organ malignancies can be observed during follow-up. The most common complications in the patients we followed were portal hypertension and malignancy (Table 4).

It is known in the literature that there is an increase in the frequency of solid organ malignancies in patients with SM (for solid cancers the hazard ratio was 2.4), and this risk is higher especially in ASM [21]. In a report of cases with advSM, the frequency of solid cancer was reported as 23% [22]. In our mastocytosis group, 6 out 104 patients (5.8%) had secondary solid organ malignancies (1 hepatocellular carcinoma, 2 lung cancer, 2 breast cancer and 1 colon cancer). Three patients died due to the secondary solid tumor and its complications (Table 3).

Manifestations of bone disease include osteopenia with or without lytic lesions, osteoporosis with or without atraumatic fracture, osteosclerosis with increased bone density, and isolated lytic lesions. In the updates of the diagnostic criteria, it is stated that an osteolytic finding greater than 2 cm should be evaluated as a C finding [23]. Osteoporosis is the most frequent finding and the frequency of osteoporosis varies between 8 and 41% [14, 24]. In our group, 18 (19.35%) patients had osteoporosis, and compression fracture was observed in 4 of these patients. 28.85% of our patients had bone symptoms (wide range from osteoporosis with fragility fractures and localized bone pain to asymptomatic osteolytic/sclerotic lesions). Osteosclerosis in 8 (8.7%) of the patients, osteolytic lesions in 4 (4.35%) were detected by x-ray, while we observed these two lesions concomitantly in 3 (3.3%) patients. We observed higher tryptase and ALP values in patients with abnormal x-ray findings. ASM patients with increased bone density/osteosclerosis had higher ALP and tryptase levels [25].

CD30 is a transmembrane receptor, normally not expressed by mast cells. Recent data suggest that CD30 expression in MC is strongly associated with SM but is not found in other myeloid neoplasms [23, 26]. Therefore, it’s diagnostic value is higher than other immunohistochemically detectable molecules. CD30 positivity rate is 85% in ASM, 27% in ISM [26]. We did not perform CD30 analysis in our patients by flow cytometry and it was examined immunohistochemically. CD30 was positive in 24 of 43 patients (13 ISM, 2 SSM, 3 ASM, 3 SM-AHN, 3 MCL). CD30 positivity was more frequently detected in our patients with hepatomegaly (6 of 24 patients, %25) and splenomegaly (8 of 24 patients, %33.3).

Currently the management of ISM, SSM and cutaneous mastocytosis is symptomatic therapy with H1 and H2 blockers, leukotriene receptor antagonist drugs and mast cell stabilizers. Patients with increased risk of anaphylaxis should be advised to carry a self-injectable epinephrine. In advanced forms, cytoreductive treatment should be used, aiming to reduce mast cell burden. With cytoreductive therapy, regression in organ infiltration can be achieved. We observed in our patients that spesific cutaneous lesions disappeared with cytoreductive therapy, especially with midostaurin and cladribine, in patients with advanced systemic mastocytosis.

The patients with ASM have been treated with cladribine or interferon-alpha ± steroids prior to the approval of midostaurin. Good response rates are observed with the use of avapritinib [27, 28]. Imatinib should be considered as a therapeutic option in the absence of a KIT D816V mutation, especially in the treatment of well differentiated bone marrow mastocytosis [3]. Hematopoietic stem cell transplantation after myeloablative conditioning regimen is a favorable therapy in eligible patients with ASM and SM-AHN [29]. Patients who initially respond well to cytoreductive therapy may benefit more from allogeneic stem cell transplantation [29, 30]. In our clinic, with cladribine therapy, we achieved good responses such as a decrease in complaints and a decrease in bone marrow infiltration before novel agents (midostaurin, avapritinib) became in to use. Midostaurin treatment was applied to 17 patients and all responded (9 CI, 8 PR). The patient who achieved a response in 3 months with avapritinib is still under treatment. Allogeneic stem cell transplantation was performed in 4 patients with diagnosis of SM-AHN or transformation to AML. It can be considered as a curative option in patients who are eligible for transplantation. In our clinic, we apply maintenance therapy with midostaurin in patients who do not have cytopenia after transplantation. Maintenance therapy may contribute to sustained response.

Several limitations of this study should be addressed. Firstly, all analyzes were made from retrospective data. Therefore, all data could not be obtained. Secondly, the genetic status of the patients could not be obtained. Most of the patients were tested for c-kit mutation, but other prognostic molecular markers (ASXL1, RUNX1, SRSF2,…) could not be obtained. CD2, CD25 and CD30 could not be measured with flow cytometry in all patients. The type of cytoreductive therapy applied was not defined according to the disease subtype. These constitute the main limitations. However, it is very important to have a multidisciplinary approach in a rare disease.

Patients with ISM have a nearly normal life expectancy. The disease progression rate is also very low, %1.7 in 5 years [14]. Risk factors for predicting transformation in this group are the presence of c-KIT mutation and an increased serum β2-microglobuline level [14]. On the other hand, advanced SM displays a poor prognosis with a median overall survival (OS) of 2–31, 24–85 and 41 months for patients with MCL, SM-AHN and ASM, respectively [15, 16]. Advanced age, weight loss, thrombocytopenia, hypoalbuminemia, and excess bone marrow blasts are known as independent adverse prognostic factors for survival [14, 16]. Survival of patients with CM was better than ISM [31]. Similarly, no patient died due to CM in our group. Of the 16 patients who died during the follow-up, 15 were diagnosed with advanced systemic mastocytosis and one was diagnosed with ISM-CML (because of secondary solid organ malignancy, lung cancer). Other major causes of death were infection, transplant-related complication, heart failure and leukemic transformation (Table 3). Regardless of the subtype, survival was observed to be significantly lower in the presence of complications (225.12 ± 12.18 vs 78.82 ± 17.67 months, *p* < 0.001), in the presence of concomitant dysplasia (212.63 ± 15.62 vs 50.59 ± 12.24 months, *p* **<** 0.001) and when the infiltration rate of mast cells in the bone marrow was above 25% (194.58 ± 14.72 vs 58.17 ± 10.68 months, *p* < 0.001) (Table 6). In the subgroup analysis, survival in cutaneous mastocytosis was better than in the other groups, as expected (Fig. 1a). While the survival time in ISM was 173.48 ± 5.40 months with the log rank test, it was determined as 103.83 ± 23.13 months in advanced SM (*p* < 0.001) (Table 6). Survival was lower in advanced SM patients. This was consistent with the literature [16, 17, 31–33]. According to the IPSM score, advanced SM group survivals were significantly less than other groups (Table 6). In addition to the WHO classification, the IPSM scoring system is indicative of the prognosis in this rare disease.

In conclusion, mastocytosis is a rare group of diseases that require a comprehensive evaluation and can show multisystem involvement. The wide range of complaints may cause patients to consult various clinics, with resulting mis- or underdiagnosis. Therefore, cooperation with different branches in an excellence center plays an important role in the diagnosis and treatment of the disease.

## Data Availability

The datasets used and/or analysed during the current study available from the corresponding author on reasonable request.
